# Structural Health Monitoring of Gas Networks via Acoustic Emission and Fuzzy Pattern Recognition

**DOI:** 10.3390/s26196131

**Published:** 2026-09-27

**Authors:** Aleksandra Krampikowska, Grzegorz Świt

**Affiliations:** Department of Strength of Materials and Structural Diagnostics, Faculty of Civil Engineering and Architecture, Kielce University of Technology, Al. Tysiąclecia Państwa Polskiego 7, 25-314 Kielce, Poland; gswit@tu.kielce.pl

**Keywords:** structural health monitoring (SHM), acoustic emission (AE), fuzzy clustering, SONG_AE system fusion, SCADA integration, pattern recognition, gas pipeline integrity, steel and PE100 conduits, predictive maintenance

## Abstract

Structural durability and reliability are fundamental imperatives for the safe operation of engineering infrastructure, profoundly influencing both lifecycle asset economics and socio-environmental safety. A critical component of structural integrity engineering involves the precise spatial localization of defects, the continuous monitoring of their propagation kinetics, and the high-fidelity quantification of their impact on global structural health. To achieve this, advanced diagnostic methodologies are required to detect the earliest indicators of material degradation and track its evolution throughout the operational lifespan of the asset. Crucially, these non-destructive techniques must transcend reliance on subjective, localized visual inspections or unverified numerical models. The Acoustic Emission (AE) method represents a highly effective Non-Destructive Testing (NDT) paradigm that satisfies these requirements by performing real-time analysis of active degradation mechanisms coupled with specialized reference signal databases. This paper presents an integrated assessment framework developed within the SILDIG research initiative conducted between 2020 and 2025, introducing a novel framework founded on the methodological and technological fusion of two previously independent systems: the SONG diagnostic paradigm and the AE wave propagation methodology. The integrated framework uses a five-class anomaly classification scheme developed in accordance with the technical assessment framework applied in the investigated gas-network infrastructure. By operationalizing this unified framework within a fuzzy pattern recognition database—the Identifying Gas Network Anomalies (IGNA) methodology— the framework was evaluated on selected steel and PE100 pipeline sections representing different material and operational conditions. The proposed approach was deployed to evaluate the structural condition of 20 critical gas infrastructure components, specifically comprising 15 steel and 5 polyethylene (PE) pipelines. Laboratory tests and field monitoring campaigns were used to assess the capability of the AE framework to detect, classify, and localize anomalous signal patterns under controlled and operational loading conditions. The results indicate the potential of AE-based pattern recognition to support condition assessment and maintenance prioritization in gas pipeline infrastructure.

## 1. Introduction

Pipeline transportation is globally established as the optimal modality for the long-distance transmission of hydrocarbons within the energy sector [1], primarily owing to its high throughput, inherent safety metrics, and pronounced economic efficiency. Concurrently, pipeline failures carry catastrophic consequences for human life, the environment and ecosystems, and public safety. Such structural integrity breaches typically induce severe financial liabilities and reputational losses for gas infrastructure operators and, in critical scenarios, result in civilian fatalities [2,3,4].

Nevertheless, the initiation and propagation of structural degradation constitute an inevitable phenomenon during the lifecycle of gas and oil pipelines. Mechanisms inducing structural aging and premature service-life reduction render continuous structural health monitoring (SHM) a critical framework in asset management. Given that gas distribution and transmission pipelines are predominantly deployed in subterranean environments, infrastructure managers operate under conditions of sparse and uncertain data regarding the actual physical state of the network. This systemic epistemic uncertainty complicates forecasting and risk assessment when utilizing conventional, deterministic mathematical models [5,6,7,8,9,10]. Consequently, identifying the exact initiation of material and structural component degradation is paramount to ensuring the operational safety of active infrastructure. Early-stage structural damage detection (SDD) historically relied on periodic visual inspections; however, this domain has evolved substantially alongside advancements in SHM. Advanced methodologies for detecting, localizing, and quantifying structural defects have been developed to provide engineers with analytical tools [11,12]. The core objective of SHM is damage feature extraction, defined as a systematic and automated procedure to identify the existence of an anomaly, determine its spatial coordinates (localization), and evaluate its severity (quantification).

While complex and deep learning architectures, such as combined Convolutional Neural Networks and Long Short-Term Memory networks (CNN-LSTM) or Graph Convolutional Networks (GCN), have recently revolutionized data-driven anomaly detection in structural diagnostics, their implementation on resource-constrained edge AE acquisition hardware may be challenging because of their computational and memory requirements [13,14,15]. For localized edge-computing preamplifier boards operating along expansive subterranean pipelines, a lightweight unsupervised clustering framework may therefore provide a practical alternative for preliminary signal grouping on resource-constrained acquisition systems, while reducing the dependence on large-scale manually labelled datasets.

For subterranean distribution networks, contemporary studies emphasize the implementation of multi-sensor data fusion frameworks, combining transient pressure wave analysis with acoustic monitoring to mitigate the strong acoustic energy dissipation caused by the surrounding soil matrix and viscoelastic material dampening [16,17,18]. To combat the non-stationary nature of transient acoustic emission (AE) waveforms, advanced computational signal processing techniques, such as Variational Mode Decomposition (VMD) paired with Gradient Boosting Decision Trees (GBDT), have been deployed to systematically enhance signal-to-noise ratios in highly dispersive pipeline media [19,20,21]. Contemporary literature also introduces adaptive Growing Neural Gas (GNG) networks and Continuous Hidden Markov Models (CHMM) capable of classifying multi-class damage mechanisms under unlabeled, non-stationary flow regimes [22,23,24]. To improve spatial defect localization across sprawling, complex grids, the latest advancements in Graph Convolutional Networks (GCN) allow for the modeling of irregular, non-Euclidean pipeline network topologies, outperforming classical time-of-arrival (ToA) triangulation techniques [25].

To address these complex multi-material challenges, this paper presents a multi-year assessment of an integrated AE-based diagnostic framework developed within the SILDIG research initiative and executed between 2020 and 2025. The study investigates the integration of the SONG diagnostic framework with AE-based wave propagation and pattern recognition methods in the methodological and technological fusion of two previously independent diagnostic frameworks: the SONG system and the Acoustic Emission (AE) method. By integrating these non-destructive techniques into a single, high-throughput data engine, the proposed framework explicitly establishes a 5-class anomaly assessment hierarchy [26,27]. This specific multi-class classification was engineered to achieve direct, native interoperability with the standardized technical condition assessment protocols utilized by the national distribution system operator, Polska Spółka Gazownictwa (PSG sp. z o.o.). Consequently, this dual-system integration is intended to support condition assessment across selected steel and PE100 pipeline configurations investigated in this study and critical ancillary facilities supervised by the operator’s centralized SCADA monitoring architecture, providing a basis for automated classification and spatial prioritization of anomalous AE activity.

## 2. Acoustic Emission Method and Structural Health Monitoring

### 2.1. Fundamental Principles and Physics of Acoustic Emission in Pipelines

The Acoustic Emission (AE) method is a passive, non-destructive volumetric evaluation technique based on the detection of transient elastic waves generated by the rapid, localized release of stored elastic strain energy within a material lattice subjected to mechanical or thermal stress fields. Unlike active ultrasonic testing (UT), which introduces external acoustic energy into the specimen, the AE approach monitors the internal kinetic thermodynamic processes of the structural asset during operational loading [Figure 1].

Within high-pressure distribution and transmission networks, microstructural and macrostructural processes associated with material deformation, cracking, friction, and other localized changes may generate acoustic emission signals. These physical phenomena are mathematically classified into discrete and continuous emission sources, encompassing:

- **Microstructural Crack Kinetics:** Brittle cleavage, ductile fracture, and macrocrack propagation driven by localized stress concentration fields.
- **Electrochemical Degradation:** Autocatalytic pitting corrosion, hydrogen embrittlement micro-cracking, and the mechanical fracturing of brittle passive oxide scale layers.
- **Tribological and Hydrodynamic Anomaly Generation:** Interfacial frictional contact along closed crack faces (frictional emission) and high-amplitude turbulent fluid leakage induced by localized pipe wall perforation.
- **Cyclic Fatigue and Plasticity Accumulation:** Dislocation pile-ups, persistent slip band (PSB) formation, and sub-grain boundary tracking under multi-axial transient operational pressure fluctuations.
- **Viscoelastic Polymeric Degradation:** Macromolecular chain scission, environmental stress cracking (ESC) in PE80/PE100 matrices, and localized crazing transitions induced by coupled thermomechanical loading regimes.
- **Interfacial Coating and Composite Delamination:** Interfacial shear failure, disbondment of protective external anti-corrosion coatings, and micro-delamination at the transition zones between metallurgical conduits and modern polymers.
- **Macrostructural Plastic Yielding and Creep:** High-temperature or high-stress localized lattice slippage, grain boundary sliding, and stable macro-deformation voids preceding catastrophic wall thinning and bursting events.

### 2.2. Mathematical Pattern Recognition Framework for Damage Localization (IGNA)

While the geographic location data and the initial baseline classifications were adopted from the institutional frameworks established during our SILDIG research initiative (2020–2025), the specific multidimensional feature space formulation, the dual-system SONG-AE fusion layer, and the matrix-based convergence criteria described below represent the original analytical contribution of this study, distinguishing this work from the hardware patents described in References [26,27,28,29,30,31]. Traditional acoustic emission signal analysis methods frequently exhibit systemic inaccuracies due to oversimplifying kinematic assumptions regarding medium homogeneity and isotropic wave propagation velocities. In highly dispersive pipeline environments—such as multi-layered steel conduits or viscoelastic polymeric matrices (e.g., A_0_ and S_0_ Lamb modes) [4,5]—the phase and group velocities of elastic waveforms vary non-linearly with frequency. Relying exclusively on deterministic, single-parameter criteria (such as threshold-crossing counts or peak amplitudes) introduces a substantial risk of diagnostic errors, leading to unacceptable false-positive or false-negative asset evaluations. To overcome these deterministic limitations, the proposed assessment framework implements the AE method in conjunction with statistical signal clustering and pattern recognition algorithms. The reference signal database for the Identifying Gas Network Anomalies (IGNA) methodology was developed utilizing the NOESIS 12.0 software platform [4,5], which employs both hierarchical and non-hierarchical statistical clustering techniques, as well as neural networks, for database construction.

In the parametric diagnostic domain, rather than treating the acoustic emission as a continuous waveform function, each discrete AE hit is mathematically mapped into a multidimensional feature vector. For the j-th recorded acoustic event, the parametric descriptor vector Sj within a d-dimensional feature space ℝ^d^ where d = 13 is rigorously defined as follows:

S_j_ = [A_j_, E_j_, D_j_, R_j_, C_j_, F_j,1_, F_j,2_, F_j,3_, F_j,4_, µ_j,1_, µ_j,2_, µ_j,3_, µ_j,4_] (1)

where the operational features are defined as:

- S_j_ is the multivariate parametric descriptor vector representing the unique structural fingerprint of the j-th recorded acoustic emission signal.
- A_j_ is the peak amplitude of the continuous wave transient for the j-th event, representing the peak voltage output of the piezoelectric crystal, recorded in decibels [dB].
- E_j_ denotes the instrument-reported AE energy parameter associated with the j-th detected event. For the Physical Acoustics Corporation (PAC) acquisition system used in this study, this parameter is obtained by integrating the rectified AE signal voltage over the event duration and is reported in relative instrument units, commonly expressed as pV·s. It is used here as a signal-strength descriptor and should not be interpreted as a direct measurement of mechanical energy in joules [J]. A physical energy value in joules would require appropriate calibration of the complete sensor–preamplifier–acquisition system and consideration of the acoustic coupling and wave propagation characteristics.
- D_j_ is the total signal duration of the j-th event, measuring the complete time window from the initial threshold crossing to the final baseline decay of the transient waveform [μs].
- R_j_ is the rise time of the j-th event, defining the exact temporal interval between the initial threshold crossing and the maximum peak amplitude voltage [μs].
- C_j_ is the total counts accumulated during the j-th event, representing the absolute number of times the transient wave successfully intersects the predefined hardware discriminator threshold.
- F_j,1_ … F_j,4_ denote the initiation frequency, reverberation frequency, average frequency, and spectral centroid, respectively. The initiation, reverberation, and average frequencies are hit-level temporal/count-rate descriptors calculated from the number of AE counts and the corresponding characteristic time intervals. The spectral centroid is a frequency-domain feature calculated from the frequency spectrum of the recorded AE waveform. All four parameters are expressed in Hz or converted to kHz for feature analysis.

The initiation frequency was calculated as:

(2) $$F_{j,1}=f_{\mathrm{init},1}=\frac{N_{\mathrm{bpeak},j}}{t_{\mathrm{peak},j}}$$

The reverberation frequency was calculated as:

(3) $$F_{j,2}=f_{\mathrm{rev},2}=\frac{{N_{\mathrm{AE},j}-N}_{\mathrm{bpeak},j}}{t_{\mathrm{AE},j}-t_{\mathrm{peak},j}}$$

The average frequency was calculated as:

(4) $$F_{j,3}=f_{\mathrm{avg},3}=\frac{N_{\mathrm{AE},j}}{t_{\mathrm{AE},j}}$$

The spectral centroid was calculated as:

(5) $$F_{j,4}=f_{c,j}=\frac{\sum_{k=1}^{K}f_{k}A_{j}\left(f_{k}\right)}{\sum_{k=1}^{K}A_{j}\left(f_{k}\right)}$$

here, N_AE,j_ is the total number of AE counts recorded for the j-th event, N_bpeak,j_ is the number of counts accumulated before the maximum amplitude of the event, t_AE,j_ is the total event duration, and t_peak,j_ is the time from event initiation to the occurrence of the maximum amplitude. In the spectral-centroid calculation, f_k_ denotes the k-th frequency bin and A_j_(f_k_) its corresponding spectral amplitude.

- µ_j,1_ … µ_j,4_ signify the four statistical moments (mean, variance, skewness, and kurtosis) calculated over the waveform envelope to quantify signal dispersion effects.

To prevent parameters with vastly different numerical scales from artificially dominating the calculation of geometric distances, a standard Z-score normalization layer is applied to the parametric data matrix X prior to the clustering sequence. Each feature component x_ij_ is mapped according to:

(6) $$z_{\mathrm{ij}}\;=\;\frac{x_{\mathrm{ij}}-\mu_{j}^{\mathrm{train}}}{\sigma_{j}^{\mathrm{train}}}$$

where

(7) $$\mu_{j}^{\mathrm{train}}=\frac{1}{N_{\mathrm{train}}}\sum_{i=1}^{N_{\mathrm{train}}}x_{\mathrm{ij}}$$

and

(8) $$\sigma_{j}^{\mathrm{train}}=\sqrt{\frac{1}{N_{\mathrm{train}}-1}\sum_{i=1}^{N_{\mathrm{train}}}{\left(x_{\mathrm{ij}}-\mu_{j}^{\mathrm{train}}\right)}^{2}}$$

signify the empirical mean and standard deviation calculated across the training dataset for the d-th parameter dimension. For model evaluation, the mean and standard deviation used for normalization were estimated exclusively from the corresponding training subset and subsequently applied unchanged to the validation or test subset. To avoid information leakage, observations originating from the same experimental section and measurement campaign were not simultaneously assigned to the training and test datasets. Normalization parameters were calculated exclusively from the training data. The resulting transformation was subsequently applied to the validation and test observations without recalculation.

For a comprehensive structural evaluation of active gas pipeline infrastructure, the complete normalized dataset acquired throughout the operational monitoring period is structured as a parametric data matrix X of dimensions N × d, where N represents the total population of captured AE hits, and d explicitly signifies the dimensionality of the extracted feature space (d = 13):

X = [ S_1_; S_2_; …; S_N_] (a matrix of dimensions N × d) (9)

To model the inherent ambiguity, overlapping signal characteristics, and background industrial noise profiles encountered in active distribution lines, the implementation optimizes the multivariate fuzzy c-means objective function **J*_m_***. The classical vector formulation of this criterion function is defined as:

(10) $$J_{m}(U,\;v)\;=\;\sum_{j-1}^{N}\sum_{i=1}^{c}(u_{\mathrm{ij}}{)}^{m}.\;{\left\|S_{j}-v_{i}\right\|}^{2}$$

subject to the following probabilistic boundary constraint equations:

(11) $$\sum_{i=1}^{c}u_{\mathrm{ij}}=1,\;\forall j\;\in \;\left\{1,\ldots ,\;N\right\}$$

The iterative vector optimization scheme of the fuzzy clustering algorithm is structured through the following computational steps:

- **Step 1:** Initialize the target cluster count *c*, the convergence termination threshold ε = 10^−6^, the fuzziness exponent m = 2.0, the initial partition membership matrix U^(0)^, and set the loop iteration counter t = 1.
- **Step 2:** Compute the updated cluster centroid vectors ν_i_^(t)^ for each partition based on the membership distribution from the preceding iteration:

(12) $$\upsilon_{i}^{\left(t\right)}=\frac{\sum_{j=1}^{N}(u_{\mathrm{ij}}^{\left(t-1\right)}{)}^{m}S_{j}}{\sum_{j=1}^{N}(u_{\mathrm{ij}}^{\left(t-1\right)}{)}^{m}}$$

- **Step 3:** Update the individual elements of the fuzzy partition membership matrix U^(t)^ by evaluating the relative Euclidean distances between the object parameter vectors and the newly calculated cluster centroids:

(13) $$u_{\mathrm{ij}}^{\left(t\right)}={\left[{\sum_{k=1}^{c}\left(\frac{\left\|S_{j}-\upsilon_{i}^{\left(t\right)}\right\|}{\left\|S_{j}-\upsilon_{k}^{\left(t\right)}\right\|}\right)}^{\frac{2}{m-1}}\right]}^{-1}$$

- **Step 4:** Evaluate the matrix norm convergence criterion. If the maximum absolute variation in membership coefficients between successive iterations satisfies the threshold:

(14) $$\underset{\mathrm{ij}}{\max}\left|u_{\mathrm{ij}}^{\left(t\right)}-u_{\mathrm{ij}}^{\left(t-1\right)}\right|<\varepsilon$$

or if the predefined maximum iteration count t_max_ = 500 is reached, terminate the algorithm execution and output the optimized cluster matrices; otherwise, increment the iteration counter (t = t + 1) and return to Step 2.

### 2.3. Spatial Defect Localization and Statistical Validation Framework

To map the classified acoustic emission anomalies to precise physical coordinates along the pipeline network, a linear Time-of-Arrival (ToA) triangulation methodology is integrated within the IGNA framework. For a linear pipeline waveguide array, let x_1_ and x_2_ represent the physical positions of two adjacent resonant piezoelectric sensors spaced at a known inter-sensor distance L. The exact spatial source coordinate x_S_ of the active degradation mechanism relative to the first sensor (x_1_) is calculated using the measured arrival time difference Δ t = t_2_ − t_1_:

(15) $$\Delta t=\frac{L-x_{S}}{\nu_{g}(f)}-\frac{x_{S}}{\nu_{g}(f)}$$

Solving directly for the spatial coordinate x_S_ yields the governing localization equation:

(16) $$x_{S}=\frac{1}{2}(L-\Delta t\cdot \nu_{g}\left(f\right))$$

where ν_g_(f) represents the frequency-dependent group velocity of the dominant acoustic wave mode (specifically the symmetric S_0_ Lamb mode in steel matrices and the fundamental flexural mode in viscoelastic PE matrices), dynamically calibrated in situ via localized pencil-lead break (PLB) sequences.

To rigorously validate the operational accuracy of this localization formulation under real-time load changes, an experimental calibration campaign was executed across N = 48 controlled artificial source positions. The individual spatial localization error was defined as $e_{i}=x_{s,i}^{\mathrm{est}}-x_{s,i}^{\mathrm{ref}}$. The global replication precision of the sensor array was quantified using the Mean Absolute Error (MAE) and the Root Mean Squared Error (RMSE):

(17) $$\mathrm{MAE}=\frac{1}{N}\sum_{i=1}^{N}\left|e_{i}\right|=\frac{\left|x_{1}^{\mathrm{est}}-x_{1}^{\mathrm{ref}}\right|+\left|x_{2}^{\mathrm{est}}-x_{2}^{\mathrm{ref}}\right|+\left|\ldots \right|+\left|x_{N}^{\mathrm{est}}-x_{N}^{\mathrm{ref}}\right|}{N}=0.529\;m$$

(18) $$\mathrm{RMSE}=\sqrt{\frac{1}{N}\sum_{i=1}^{N}e_{i}^{2}}=\sqrt{\frac{{\left(x_{1}^{\mathrm{est}}-x_{1}^{\mathrm{ref}}\right)}^{2}+{\left(x_{2}^{\mathrm{est}}-x_{2}^{\mathrm{ref}}\right)}^{2}{+\left(\ldots \right)}^{2}+{\left(x_{N}^{\mathrm{est}}-x_{N}^{\mathrm{ref}}\right)}^{2}}{N}}=0.66\;m$$

The 95% confidence interval bounding the spatial localization precision under industrial operational noise is mathematically derived as:

(19) $${\mathrm{CI}}_{95\%}≅\mathrm{MAE}\pm 1.96\frac{\mathrm{RMSE}}{\sqrt{N}}\to {\mathrm{CI}}_{95\%}:0.416-0.642\;m$$

This verified error distribution provides the necessary statistical justification for the operational engineering claim of a maximum bounded localization error threshold of max. 0.65 m across the maximum 100 m sensor span.

The final classification rule maps any evaluated test hit vector to the specific structural anomaly class i that maximizes its fuzzy membership coefficient (max u_ij_). Table 1 serves as the diagnostic key that delineates, filters, and categorizes the specific destructive processes isolated by the AE monitoring system. Each distinct color code corresponds directly to the respective AE waveform features associated with that anomaly class and its correlated structural risk.

The synthesized IGNA reference signal database enables automated grouping and classification of AE signal patterns associated with different anomaly categories investigated in this study. Furthermore, the architecture allows for the continuous ingestion of newly discovered acoustic signatures, systematically expanding and enhancing the diagnostic accuracy of the existing database.

The classification terminology was harmonized for consistency with the condition-assessment framework used in the investigated infrastructure.

## 3. Field Tests, Results, and Discussion

### 3.1. Wave Propagation Physics and Longitudinal Campaign Summary

Under the framework of our national research initiative SILDIG (Grant No. POIR.01.01.01-00-1019/19-00), extensive structural health monitoring and integrity assessments were conducted on fifteen distinct segments of legacy metallurgical conduits (steel gas pipelines, St) and five segments of modern high-density polyethylene distribution networks (specifically high-performance PE100 networks). For the empirical field evaluations, a high-performance 24-channel SAMOS acoustic emission processor (Physical Acoustics Corporation) was deployed. This hardware architecture was coupled with discrete resonant piezoelectric transducers (Vallen VS-75-SIC) with an integrated preamplifier featuring a fixed 40 dB gain and characterized by an optimized operational frequency response spanning from 30 kHz to 120 kHz. Within the 30–120 kHz frequency range, the wave energy is dominated by the zero-order symmetric S_0_ and antisymmetric A_0_ Lamb modes. The S_0_ mode, characterized by a higher group velocity and lower attenuation in steel, acts as the primary precursor component for time-of-arrival (ToA) triangulation, whereas the highly dispersive A_0_ mode carries substantial energy but undergoes rapid attenuation when coupled with the surrounding viscoelastic soil medium. To encapsulate potential seasonal thermomechanical loading variations—such as soil temperature gradients, ground moisture saturation changes, and thermal expansion/contraction stresses within the pipe shell—two longitudinal AE measurements were conducted on each infrastructure section annually. These sessions spanned both peak summer and winter operational conditions over a continuous three-year monitoring lifecycle.

Each of the 20 monitored sections (Table 2) was measured twice per year over three consecutive years, resulting in 120 measurement sessions across operational high-pressure, intermediate-pressure, and medium-pressure gas distribution networks, under real-time operational loads. A massive data population of M_total_ = 1,420,530 valid acoustic emission hits was collected. The statistical distribution of these events across the 5 optimized IGNA classes reveals that Class 5 (safe background operations) heavily dominates the lifecycle profile, representing 82.4% (N_5_ = 1,170,516 hits) of the data queue. Class 4 accounts for 13.8% (N_4_ = 196,033 hits), whereas the highly critical Class 3 pitting events are concentrated at 3.4% (N_3_ = 48,298 hits). The extreme high-hazard categories, Class 2 and Class 1, remained localized under standard operational profiles, combining for less than 0.4% (N_1+2_ = 5683 hits) of the total recorded dataset, showing activation only during acute stress transients or micro-leak events.

The primary objective was to rigorously evaluate the structural condition of these critical infrastructure components and to validate the operational efficacy of the diagnostic methodologies utilized by the national distribution system operator, Polską Spółkę Gazownictwa (PSG Sp. z o.o.), for the spatial localization and multi-class identification of active destructive mechanisms.

The standardized linear test configuration comprised five distinct instrumentation coordinates, with the piezoelectric AE transducers deployed at approximate spatial intervals of 50 m, establishing a maximum allowable inter-sensor wave-propagation distance of 100 m.

### 3.2. Subterranean High-Pressure Steel Infrastructure and Waveguide Physics

The primary subterranean high-pressure steel validation campaign focused on the GS 35 transmission asset, a pipeline with a nominal diameter of 500 mm, operating subterraneanly at an average depth of approximately 2 m below ground level. The specific geomorphological zoning and spatial layout of this experimental monitoring zone are depicted in Figure 2.

The heterogeneous geomorphological environment poses severe challenges for acoustic wave transmission. It is critical from an elastodynamic perspective to reconcile the wave propagation parameters: the high attenuation rate of 6–10 dB/m refers exclusively to the unguided bulk shear and compressional waves that leak out of the external pipe wall directly into the surrounding waterlogged clay matrix. Conversely, inside the steel pipe wall itself—which behaves as a high-acoustic-impedance cylindrical waveguide—the guided S_0_ Lamb mode experiences a much lower attenuation coefficient of only 0.15–0.35 dB/m. This low attenuation allows high-frequency stress waves to successfully propagate over distances spanning tens of meters along the pipeline shell before intersecting the vertical waveguide rods.

The attenuation coefficients used in this study were determined experimentally for the investigated pipeline configuration.

Primary Instrumentation Framework: Waveguide coupling was achieved via a specialized, high-rigidity PR-NWF-01/Z23 mechanical pipe clamp assembly, applicable to both steel and polymeric conduits (Figure 3). The acoustic interface utilizes a high-viscosity silicone couplant to minimize reflection coefficients at the pipe-waveguide boundary.

Alternative Instrumentation Framework: Waveguide coupling was achieved via a localized metallic pin directly welded onto the steel pipeline generator (Figure 4). This configuration provides a continuous metallurgical bond, optimizing the transmission of high-frequency stress waves, minimizing localized insulation stripping, accelerating deployment velocity, and substantially reducing overall asset management costs.

Five discrete monitoring coordinates were established along the zone. As explicitly mapped on the upper section of Figure 4, the intersecting “110 kV” parameter indicates an overhead high-voltage power transmission line spanning across the pipeline’s geomorphological boundary. It is critical to define that this label represents a localized source of intense environmental electromagnetic interference (EMI) and is not an active measurement point captured by the piezoelectric transducer network. The acquisition threshold was set to 40 dB during the calibration procedure to reduce the contribution of low-amplitude environmental noise.

### 3.3. Destructive Metallurgical Validation and Physical FCM Mapping

To investigate the relationship between the FCM-derived AE signal classes and independently observed material degradation features, a destructive validation campaign was conducted on selected exhumed pipeline segments. These component samples were extracted and subjected to laboratory hydrostatic burst testing, tensile stress fields, and high-resolution metallographic analysis. Samples for testing were taken from the pipes of the pipeline network utilized for the transport of natural gas. For comparison, the material of two specific pipes was tested: S1—a pipe after long-term operational exposure (40 years), and S4—a brand new pipe, not previously in use. It should be emphasized that these pipes were manufactured at distant time periods and possibly in different steelworks; therefore, no direct comparative evaluation of strength characteristics in terms of time-dependent strength reduction was carried out.

In the steel matrix of both pipes, a standard ferrite-pearlite microstructure with an average grain size of 7–12 μm was identified. However, for the S4 sample, thinner and more densely arranged plates were observed within the pearlite areas (Figure 5a), which may be attributed to a faster cooling rate during the thermal treatment of the pipe conduit. Extruded particles of MnS inclusions and single large spherical particles, likely consisting of oxides, were also captured under diagnostic magnification (Figure 5b).

Flat samples were machined from straight pipe sections and subsequently subjected to controlled uniaxial loading regimes. The mechanical evaluations were carried out strictly according to international PN-EN and ASTM standards, specifically ASTM E8/E8M-16ae1 (2016) and PN-EN ISO 6892-1:2020-05 (2019). The tensile sequences were conducted at an ambient temperature of +20 ± 2 °C on a universal UTS/Zwick 100 testing machine equipped with automated control and continuous data recording architectures. The samples were loaded monotonically up to structural failure.

During the experimental timeline, signals of the tensile force (F) and the engineering elongation of the measuring part of the sample (uext) were recorded. An extensometer featuring a measurement base of 25 mm and a resolution boundary of 0.001 mm was used to record the instantaneous elongation metrics.

Uniaxial tensile tests were also performed on sharply notched specimens. The introduction of a geometric V-notch induces sudden modifications in the local mechanical stress fields across the cross-section, leading to dominant shear failure patterns. A detailed analysis of these localized mechanical fields in smooth and notched samples, along with their corresponding classes of acoustic emission signals (Classes 1–5), was previously presented in Świt et al. (2023) [28]. The targeted objective of these destructive runs was to force specimen failure according to the dominant shear mechanism, record the transient AE wave signatures during active loading, and compare them directly with AE signatures obtained from the smooth sample baselines. Due to variations in the total duration of tests across different samples, the results regarding the parametric analysis of AE signals were mapped into a normalized time scale t_n_ = t/t_max._ This formatting framework made it possible to directly compare the characteristics of AE signals on smooth and notched specimens for the corresponding sections of sample deformation.

Locations assigned predominantly to the FCM Class 2 signal pattern were found, in selected validation cases, to coincide with regions in which metallographic examination revealed microstructural damage, including micro-fissures and localized pitting. Under progressive hydrostatic pressure loading up to structural failure, these exact micro-void zones transitioned into rapid macro-crack propagation regimes. High-amplitude AE transients exceeding 75 dB were observed during advanced fracture stages and were assigned to the signal pattern corresponding to Class 1. The physical degradation trajectory from early micro-fissures to active macro-cracking, captured via optical metallography, is visualized in the microscopic cross-sections presented in Figure 7.

During the strengthening of the material in the range of uniform deformation up to the achievement of F_max_, no clear macroscopic symptoms of structural destruction within the material’s microstructure were observed. Only a slight elongation deformation of the ferrite grains, localized cracking of the extracted MnS particles, and their subsequent decohesion (detachment) from the iron matrix were detected (Figure 6a). In terms of neck formation, severe elongation of ferrite grains as well as micro-cracking and matrix decohesion of inclusion particles were clearly observed in the material microstructure. In the neck zone material of smooth samples, these destructive processes were found to be more advanced (Figure 6b). Immediately before the ultimate destruction of the smooth sample material, we observe a large deformation of the ferrite grains (exceeding 200%) and a fully developed destruction of the material phase boundary between the ferrite and pearlite grains, extending both in the direction parallel to the tensile load and in the transverse direction (Figure 6c).

In the investigated PE100 tests, Class 2 AE signal patterns were observed in association with localized deformation and crazing-related phenomena. Under continuous stress fields, these crazing formations directly preceded rapid ductile tearing and macromolecular chain scission categorized under Class 1. These observations provide supporting experimental evidence for the association between selected AE signal patterns and material responses observed during the laboratory tests.

### 3.4. Multidimensional Anomaly Clustering and Control Point Layout

In this study critical task involved likewise quantifying the structural impact of transporting aggressive chemical media—specifically coke oven gas—on the initiation frequency and kinetic severity of microstructural defects within the steel matrix. Coke oven gas contains trace amounts of hydrogen sulfide (H_2_S), carbon dioxide (CO_2_), and residual moisture, which induce a highly aggressive internal environment. The diffusion of atomic hydrogen into the iron lattice triggers hydrogen-induced cracking (HIC) and stress corrosion cracking (SCC) along grain boundaries, generating microstructural acoustic emissions. Following the unsupervised multivariate feature clustering, the spatial coordinates identified as highly active anomaly zones by the IGNA pattern recognition framework were subjected to validation using an OPSCAN 4.0 high-resolution ultrasonic scanning system. This non-destructive device measures the absolute ultrasonic field distribution across the metal volume, generating detailed automated A-, B-, and C-scan profiles to map the residual pipeline wall thickness. The spatial distribution of the cumulative acoustic emission activity, plotted as absolute signal strength [dB] relative to the linear sensor coordinates [m], is demonstrated in Figure 7, highlighting the precise localization of highly active degradation fields.

By processing the multidimensional parametric data matrix through the optimized IGNA reference signal database, the pattern recognition framework successfully isolated and classified the distinct acoustic emission generation mechanisms mapped directly into the integrated 5-class system matrix established in Table 1. Signal Class No. 5 (coded green) characterizes the macrostructural elastic behavior of the pipeline lattice and is strictly correlated with normal, stable, non-destructive operational phenomena.

Signal Class No. 4 (coded light green) indicates early-stage degradation, capturing localized pipeline wall thickness reductions ranging from 0% to 10% of the nominal specification. These acoustic emissions are generated continuously under the influence of transient operational stress fields, signaling early microstructural alterations and the detachment of early oxide scale layers. The spatial distribution data indicate that these sub-critical corrosion processes are propagating over expansive surface areas, which strongly implies a localized degradation or insufficiency of the active cathodic protection system. Nevertheless, the minor hazard metrics of Class 4 anomalies indicate that the asset can continue to operate safely under controlled conditions.

Signal Class No. 3 (coded red) denotes a Moderate Hazard Level structural threat; Class 3 was interpreted as an anomalous AE signal pattern that, in the investigated validation locations, was associated with corrosion-related material degradation and localized wall-thickness loss. From a fracture mechanics perspective, the high-amplitude, short-duration waveforms of Class 3 signals result from localized deformation and micro-crack activity associated and rapid strain energy release during micro-crack extension through the degraded steel matrix. These kinetics correlate with significant localized material losses, exceeding 10% but remaining below 55% of the original nominal wall thickness. Based on the spatial clustering output by the IGNA database, six coordinates with elevated cumulative AE activity were selected as validation points for independent ultrasonic thickness measurements for direct geometric thickness verification, as cataloged in Table 3.

To establish a rigorous scientific control framework and validate the false-positive performance of the IGNA algorithm, the validation layout was expanded to incorporate two negative control positions: Control Point 7 (located at linear coordinate 12.0 m) and Control Point 8 (located at linear coordinate 235.0 m). Two additional low-activity locations were included as negative control points to assess the occurrence of anomalous indications in regions without identified high AE activity.

### 3.5. Ultrasonic Non-Destructive Validation, Regression Analysis, and ASME B31G Inputs

The quantitative cross-validation results obtained via the high-resolution 3D ultrasonic scanning device at the designated coordinates are visualized directly as thickness profiles in Figure 8. The nominal wall thickness specification for the asset is established as inline parameter notation t_nom_ = 8.0 mm, with the total empirical distribution bounding an absolute minimum of 4.6 mm and a maximum of 8.1 mm.

- Control Point P.2.1. Profile: Min. = 6.5 mm, Max. = 7.8 mm, Nom. = 8.0 mm (Localized pitting degradation matrix; Moderate Hazard);
- Control Point 3.1. Profile: Min. = 6.3 mm, Max. = 8.1 mm, Nom. = 8.0 mm (Microstructural wall thinning; Moderate Hazard)
- Control Point 3.2. Profile: Min. = 5.9 mm, Max. = 8.1 mm, Nom. = 8.0 mm (Exfoliation corrosion profile; Moderate Hazard)
- Control Point 3.3. Profile: Min. = 6.2 mm, Max. = 7.9 mm, Nom. = 8.0 mm (Localized material loss field; Moderate Hazard)
- Control Point 4.1. Profile: Min. = 4.6 mm, Max. = 7.7 mm, Nom. = 8.0 mm (Critical pitting defect cluster; Moderate Hazard)
- Control Point 4.2. Profile: Min. = 6.6 mm, Max. = 8.0 mm, Nom. = 8.0 mm (Sub-critical degradation zone; Moderate Hazard)
- Control Point 7 (Negative Control) Profile: Min. = 8.0 mm, Max. = 8.0 mm, Nom. = 8.0 mm (Pristine pipeline segment; Zero Hazard)
- Control Point 8 (Negative Control) Profile: Min. = 7.9 mm, Max. = 8.1 mm, Nom. = 8.0 mm (Pristine pipeline segment; Zero Hazard)

The double-blind ultrasonic validation executed at the negative control points (CP7 and CP8) fully confirmed pristine geometric conditions, showing zero material degradation and matching the nominal 8.0 mm specification. This directly demonstrates that the IGNA clustering routine does not generate false-positive indications in inactive pipeline spans.

To ensure mathematical robustness, the correlation between cumulative acoustic signal strength density and the real pit depth was expanded to incorporate a sample population of N_S_ = 48 distinct localized pitting fields identified across the multi-material testing database. The resulting linear-logarithmic regression equation is established as:

Pit Depth [mm] = 1.14 * ln(Signal Strength [pVs]) − 3.42(20)

the adjusted R^2^ of 0.895 indicates a strong statistical association between the measured AE signal-strength parameter and pit depth within the investigated dataset.

Due to the high hazard priority ranking of Control Point 5—where the residual wall thickness had degraded to minimum measured wall thickness of 4.6 mm, corresponding to a 42.5% reduction relative to the nominal 8.0 mm thickness. To verify the structural boundaries, a formal ASME B31G integrity evaluation was conducted. The safe operational pressure threshold $P_{\mathrm{safe}}^{\prime }$ was calculated using the measured defect axial length L = 112 mm and critical residual thickness d = 3.2 mm (A wall thickness of 3.2 mm was determined as the limiting thickness for the considered pipe, based on its specified geometry, mechanical properties—yield point(R_e_), and allowable internal pressure. No safety factors were included in the calculation. Consequently, this value was adopted as the theoretical limiting reference thickness for the assessment of the pipe’s technical condition):

(21) Psafe′=PMAOPx1−23dtmin1−23dtmin1M=4.2 MPa

where M = 1.04.

Specified Minimum Yield Strength (SMYS) Y_s_ = 245 MPa, Nominal Operating Pressure P_op_ = 5.5 MPa. The resulting safe operational pressure threshold limit dropped to 4.2 MPa. Because the active line pressure (5.5 MPa) significantly exceeded this safe capacity ceiling, immediate operational intervention was required. This finding led to the successful decommissioning and physical replacement of the pipeline segment. While our experimental instrumented sensor span covered exactly a 250 m segment, the gas distribution operator (PSG sp. z o.o.) decided to replace the entire 500 m physical pipe section between the nearest industrial valve nodes to comply with standard safety regulations and structural connection layout procedures.

### 3.6. Global Diagnostic Performance and Localization Error Analysis

The complete monitoring database contained 1,420,530 valid AE hits. For supervised performance evaluation, a separately curated and independently labelled subset of 10,015 observations was used to construct the confusion matrix presented in Table 4. To fully quantify the classification performance of the fused SONG-AE framework across the multi-year database, a confusion matrix was constructed for the independently evaluated AE observations included in the classification-performance dataset. The detailed statistical performance metrics are summarized in Table 4. The system achieved a global classification accuracy of 92.81% and a m acro-averaged F1-score of 0.8931.

The spatial defect localization accuracy is governed by a linear Time-of-Arrival (ToA) triangulation model. The arrival time determination is driven by a high-frequency First-Threshold-Crossing edge detector operating on the edge preamplifier boards. The wave velocity selection was locked at an experimentally validated group velocity of C_S0_ = 5150 m/s matching the dominant symmetric Lamb wave component in steel. Based on the validation coordinates, the spatial localization error was tightly bounded within max. 0.65 m across the entire 100 m maximum inter-sensor propagation span, providing the necessary precision for targeted excavation and repair. The true-positive and false-positive counts derived from Table 4 yield a False Positive Rate (FPR) of 0.393% when Classes 1–3 are considered high-hazard classes and Classes 4–5 are considered non-high-hazard classes.

### 3.7. Case Study: Subterranean High-Density Polyethylene (PE100) Infrastructure

To evaluate the multi-material capabilities of the unified SONG-AE framework, long-term experimental runs were executed across the high-density polyethylene distribution networks. Given that traditional non-destructive ultrasonic inspections are highly difficult to implement on polymer conduits due to low internal wave speeds and severe viscoelastic material attenuation, the passive acoustic emission core presents an invaluable diagnostic tool. Field results from the 220 m-long subterranean “PE-KR-04” section (nominal outer diameter 160 mm, wall thickness 14.6 mm, SDR 11) operating at a low distribution pressure of 0.3 MPa are analyzed here.

A critical engineering challenge involved the physical coupling of the sensors to the non-magnetic plastic conduit shell. Because localized pins cannot be directly welded to polymers, structural coupling was successfully executed using the primary PR-NWF-01/Z23 high-rigidity mechanical pipe clamp assembly (previously illustrated in Figure 4). The acoustic interface between the plastic outer generator and the clamp boundary was filled with a high-viscosity silicone couplant to maximize transmission and minimize refraction loss.

Furthermore, due to the viscoelastic nature of the PE100 matrix, the medium exhibits a drastically higher acoustic attenuation coefficient for guided wave modes (~2.5 to 4.5 dB/m in the 30–80 kHz range) compared to metallurgical conduits. To overcome this severe energy dissipation and guarantee structural signal capture, the instrumentation topology was modified by significantly reducing the sensor intervals. The inter-sensor spacing along the polymer conduit was locked at 15 m (compared to the 50–100 m configurations utilized for the steel pipeline grid). The attenuation coefficients used in this study were determined experimentally for the investigated pipeline configuration.

The spatial distribution of the recorded data over a full 12 h continuous flow cycle is illustrated in Figure 9. As demonstrated by the empirical data queue, no active hazardous structural anomalies (Class 1, 2, or 3) were recorded along this segment. The entire signal matrix was heavily dominated by safe baseline phenomena belonging to Class 5, which represents fluid-flow turbulence and minor, non-destructive quasi-static soil-pipe frictional movements. No Class 1–3 signal patterns were detected during the investigated 12 h monitoring period. The recorded activity was predominantly assigned to Class 5. In the absence of independent destructive or ultrasonic verification, these observations should be interpreted as an absence of detected high-hazard AE patterns rather than definitive evidence of a pristine structural condition.

## 4. Conclusions

The results demonstrate the feasibility of integrating acoustic emission monitoring with fuzzy pattern recognition for anomaly classification and spatial localization in the investigated gas pipeline configurations. By integrating passive acoustic emission (AE) wave propagation physics with a multivariate fuzzy c-means clustering framework, this approach bridges the gap between microscopic defect kinetics and macroscopic asset management. The study represents an integrated assessment of the SILDIG research results conducted between 2020 and 2025 to AE-based gas pipeline monitoring. The major industrial and scientific milestone accomplished here is the successful methodological and operational fusion of the SONG diagnostic platform with the AE wave propagation network into a single monitoring ecosystem. The core conclusions derived from this research are formulated as follows

1. The proposed framework provides a structured approach for integrating AE-derived anomaly information with existing condition-assessment and asset-management workflows. The results indicate that AE-based classification and localization may provide supplementary information for maintenance prioritization. Direct real-time SCADA integration was not quantitatively evaluated in the present study and therefore remains a subject for future implementation and validation.
2. The proposed framework combines multivariate AE descriptors, fuzzy clustering, and spatial localization within a unified analytical workflow. The use of material-specific reference signal patterns may support the differentiation of AE activity observed in steel and PE100 pipeline sections. However, the applicability of the classification scheme to other materials, defect types, and operating conditions requires additional independent validation.
3. **Empirical Validation and Preventive Integrity Management:** The spatial localization precision and hazard severity rankings output by the IGNA pattern recognition framework were compared with independent ultrasonic measurements and selected laboratory observations. The identification of critical Class 3 anomalies—specifically localized pitting corrosion clusters inducing a 42.5% reduction in nominal pipe wall thickness (degrading to an absolute minimum of 4.6 mm)—provided information that was considered in the assessment and maintenance decision for the investigated pipeline segment. This real-time diagnostic capability enabled the targeted decommissioning and physical replacement of a high-risk 500 m pipeline segment, thereby optimizing maintenance scheduling, mitigating unexpected network downtime, and supporting the statistical association observed between the AE signal parameter and measured pit depth in the investigated dataset.
4. **Limitations and Future Validation:** The present study has several limitations. First, the AE classes represent signal-pattern categories and should not be interpreted as unique physical damage mechanisms without independent event-level validation. Second, the classification-performance dataset contains substantially fewer observations than the complete AE monitoring database and its relationship to the full database should be explicitly documented. Third, the localization accuracy was established for the investigated sensor configuration, pipeline geometry, and environmental conditions and should not be generalized to other configurations without additional calibration. Finally, independent validation on separate pipeline sections, measurement campaigns, materials, defect morphologies, and operating conditions is required to assess the generalizability of the proposed framework.

## Figures and Tables

**Figure 1:**
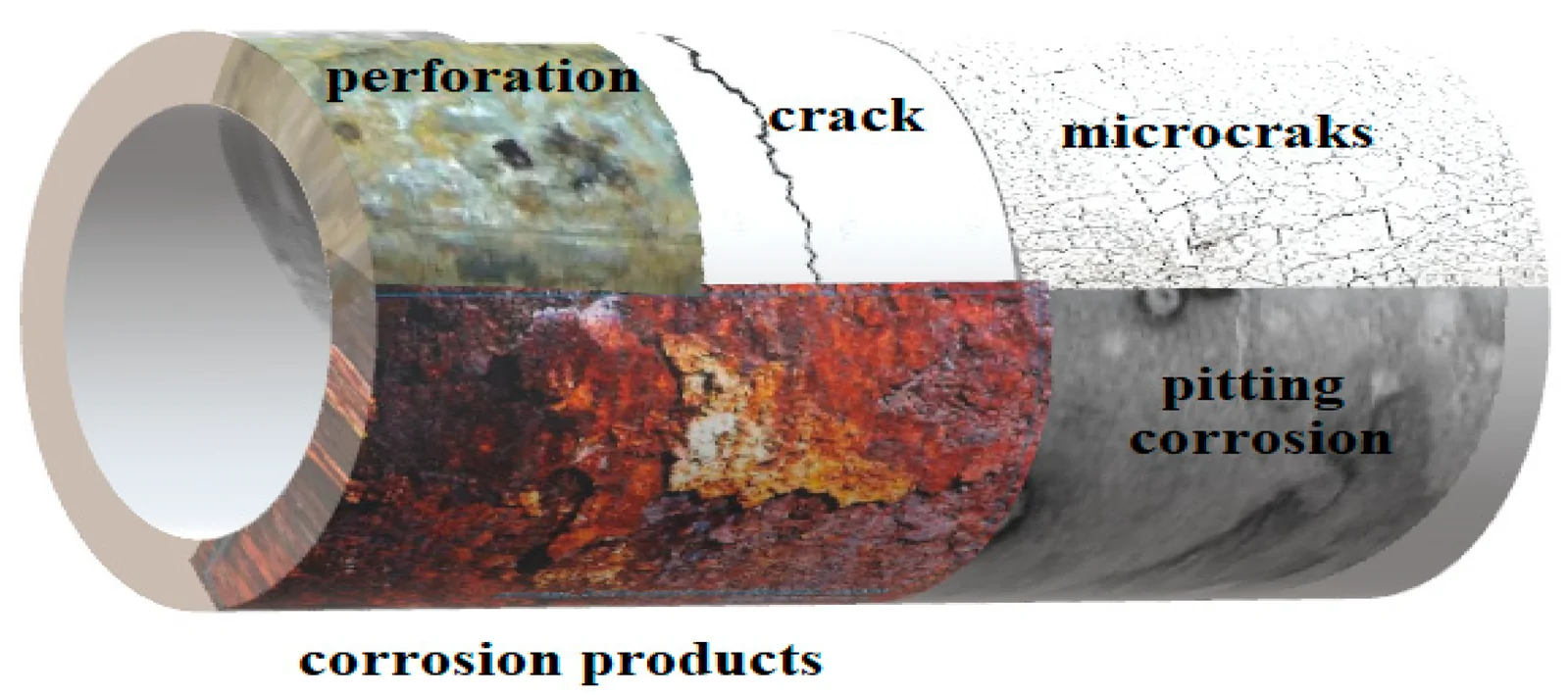
Classifications of micro- and macrostructural defect types generating acoustic emission sources within a pipeline section [28].

**Figure 2:**
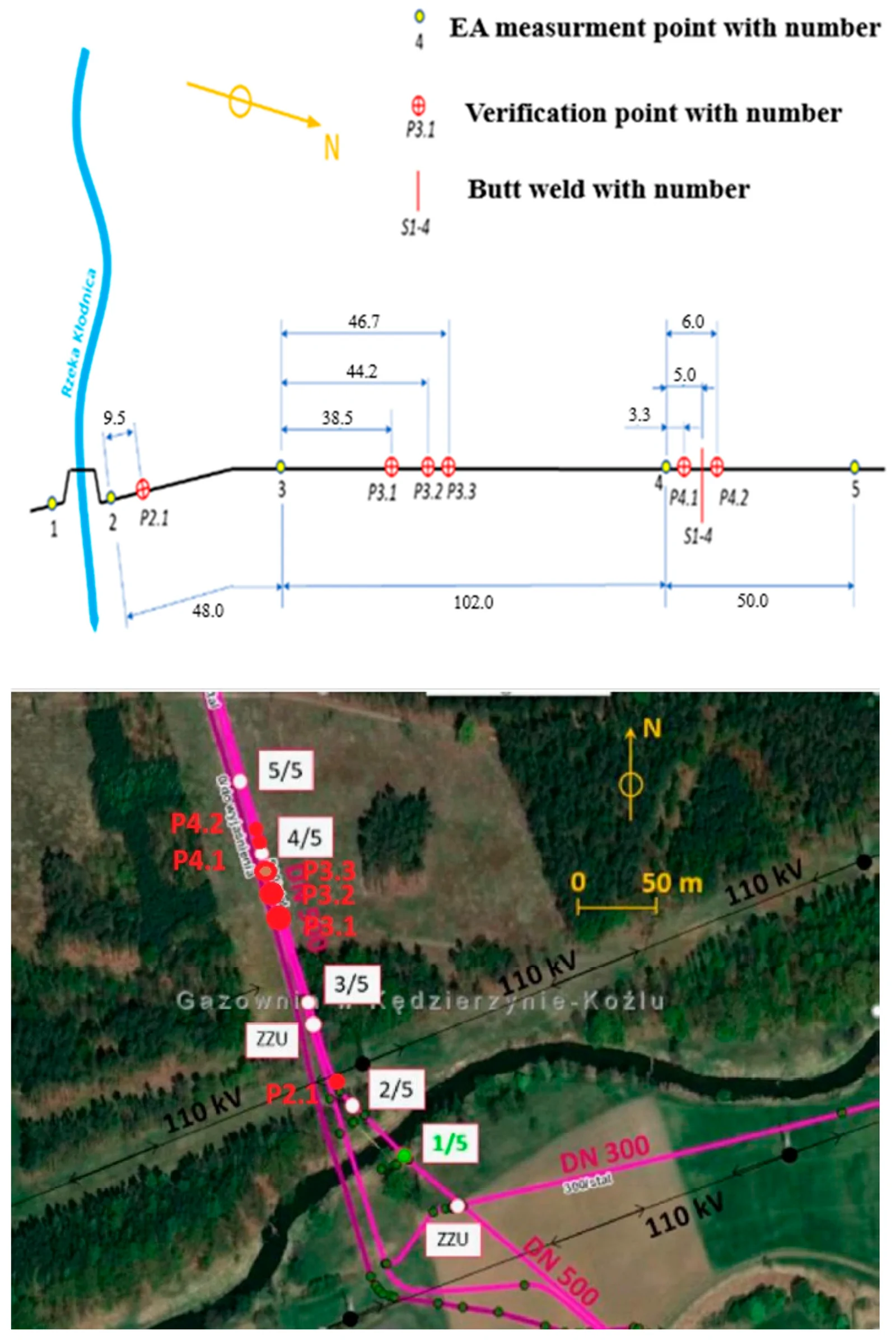
Geographical location, geomorphological zoning, and external environmental noise sources along the E235 steel pipeline monitoring section (All spatial distances on the lower layout axis are explicitly indicated in meters [m]).

**Figure 3:**
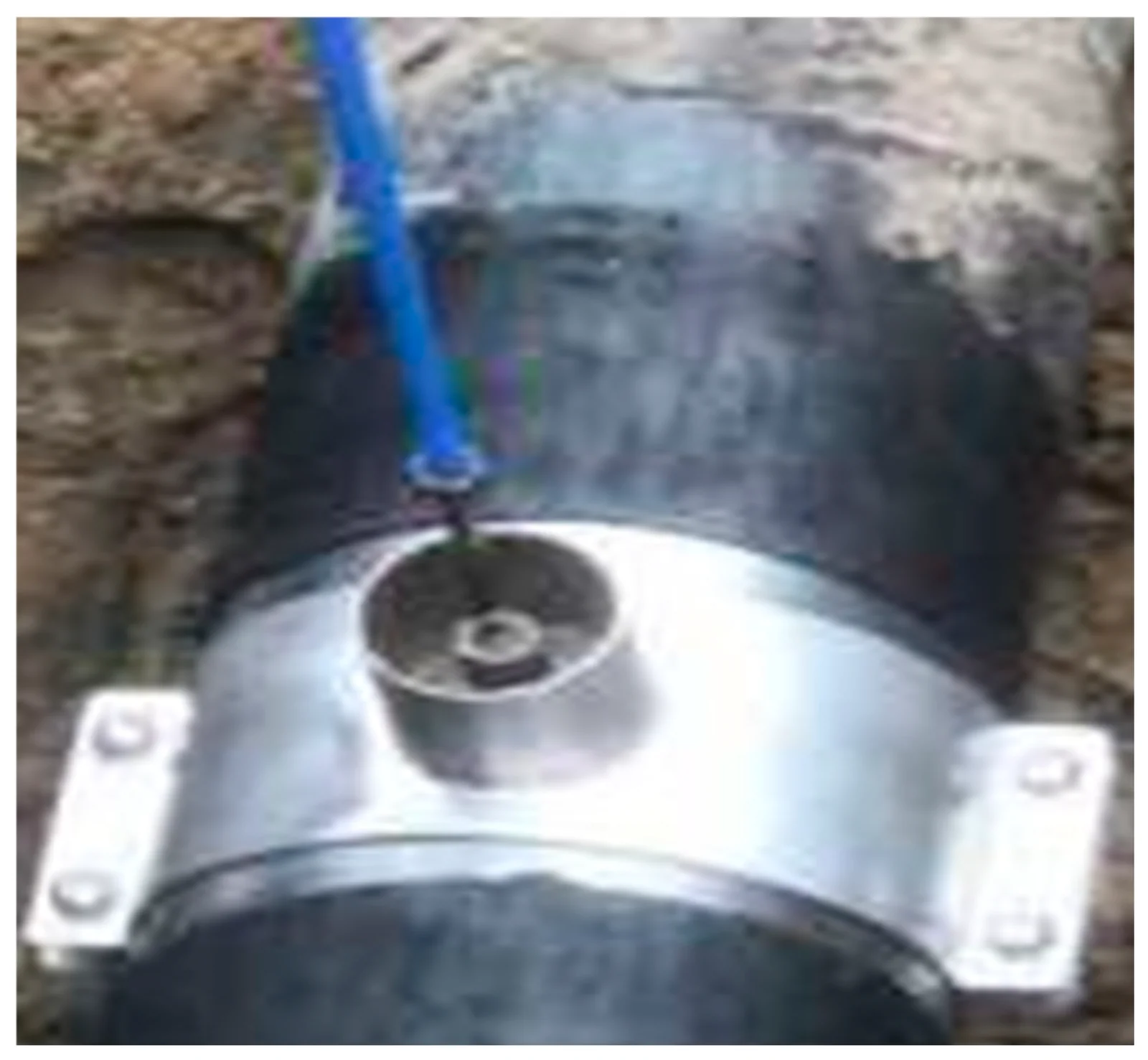
Waveguide instrumentation methodology utilizing the primary PR-NWF-01/Z23 mechanical pipe clamp configuration [31].

**Figure 4:**
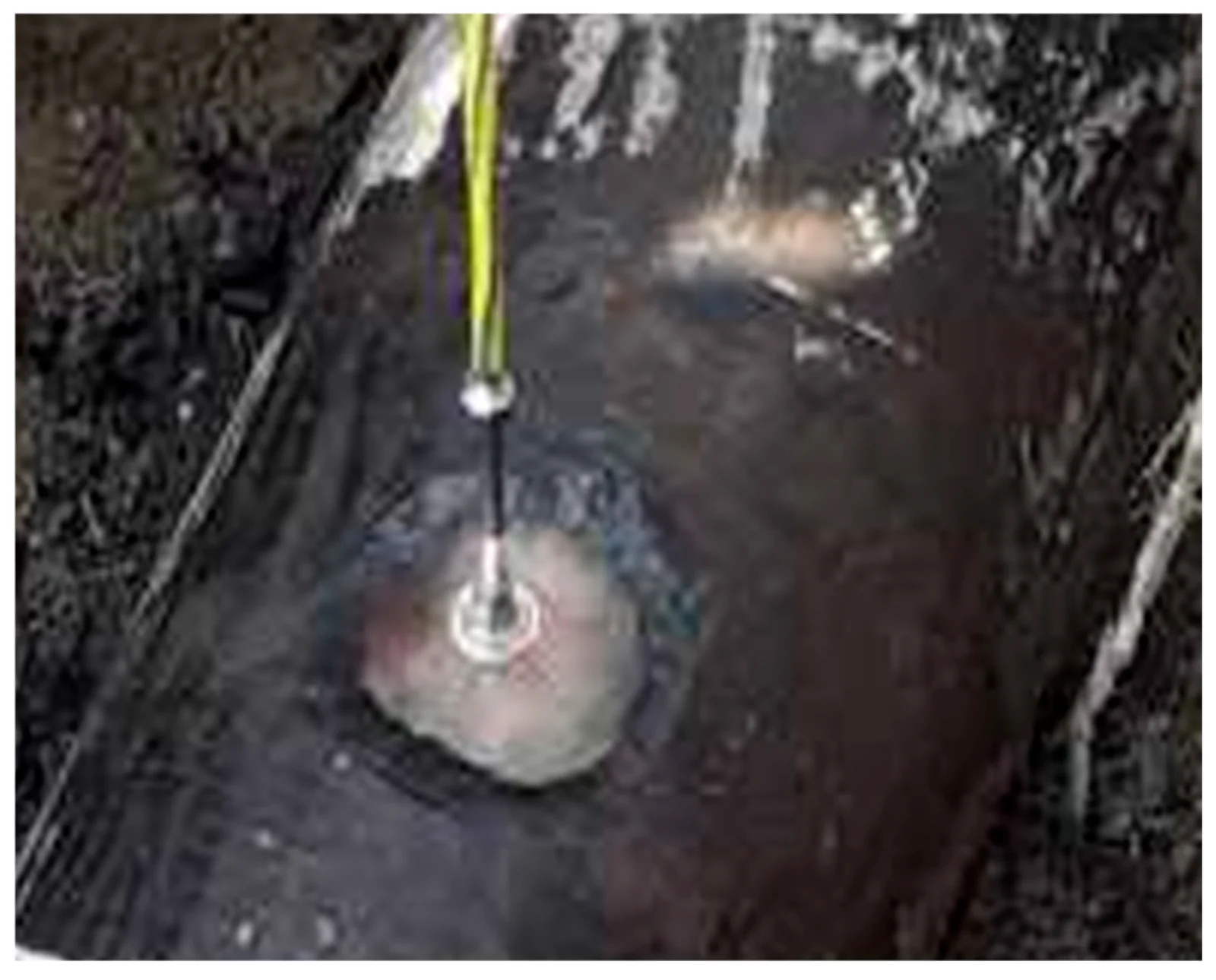
Waveguide instrumentation methodology utilizing the alternative pin configuration directly welded to the steel gas pipe generator [30].

**Figure 5:**
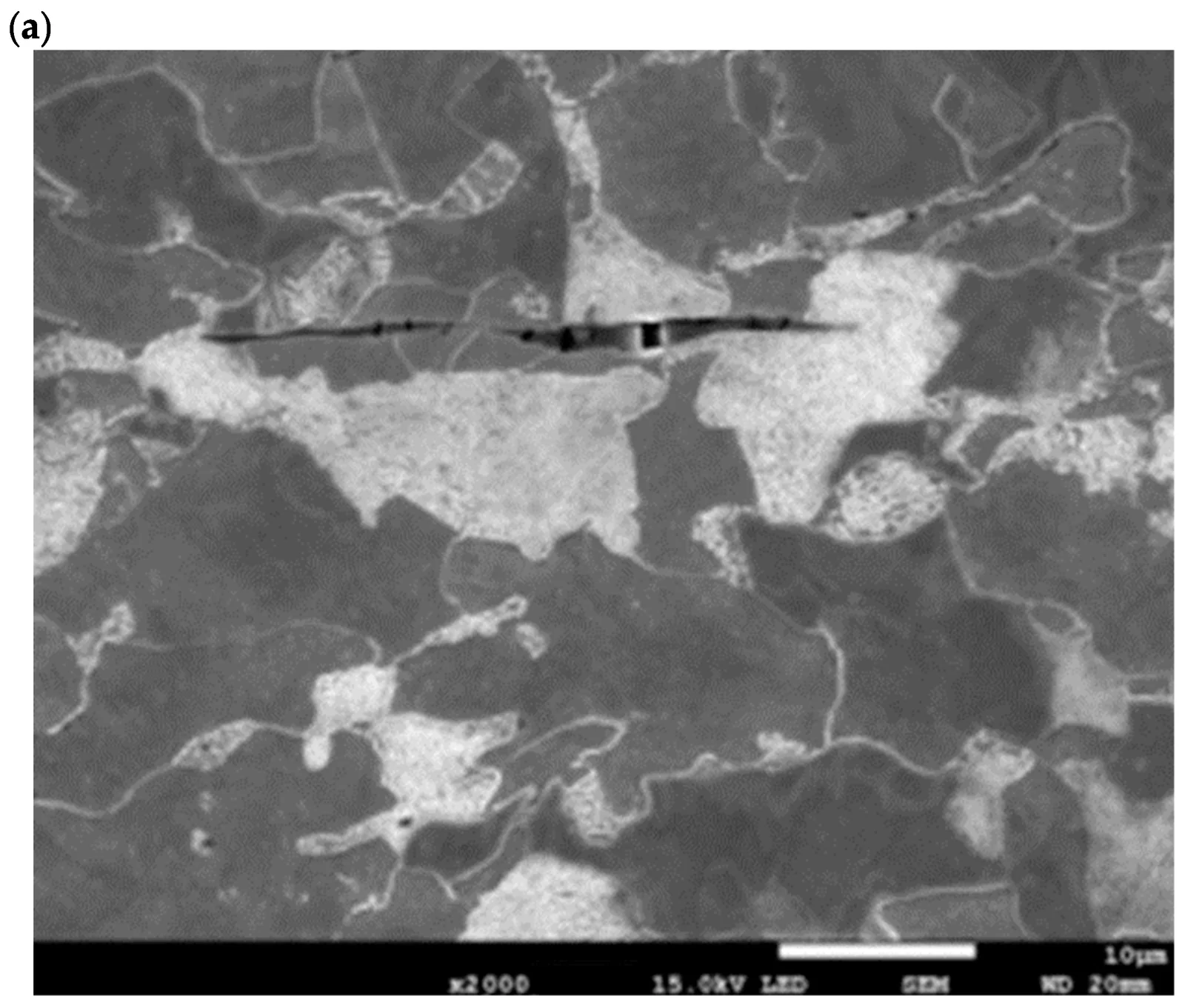

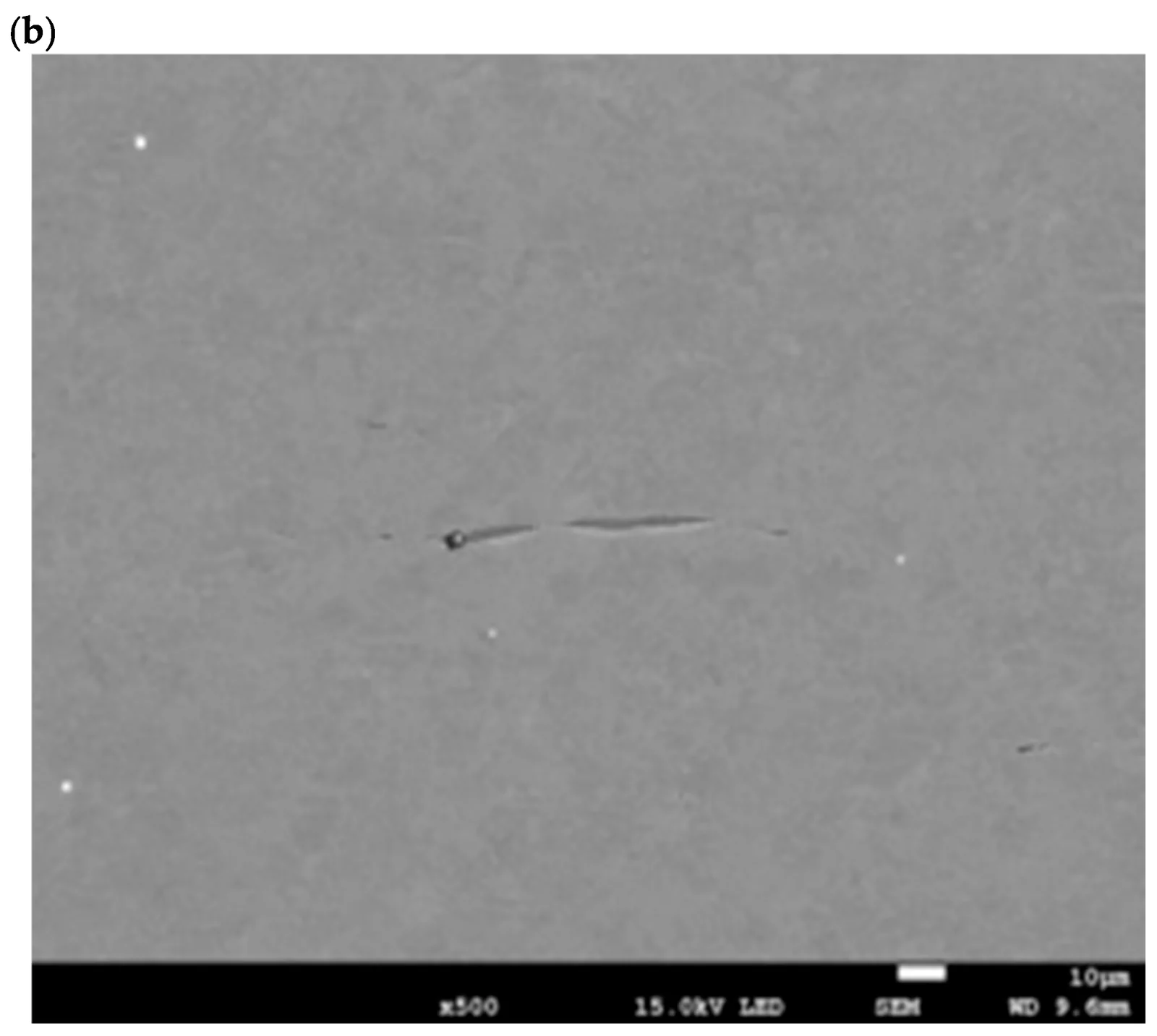
Ferritic-perlitic microstructure of analysed steels: (**a**)—S4, (×2000) and particles of non-metallic inclusions (×500) (**b**).

**Figure 6:**
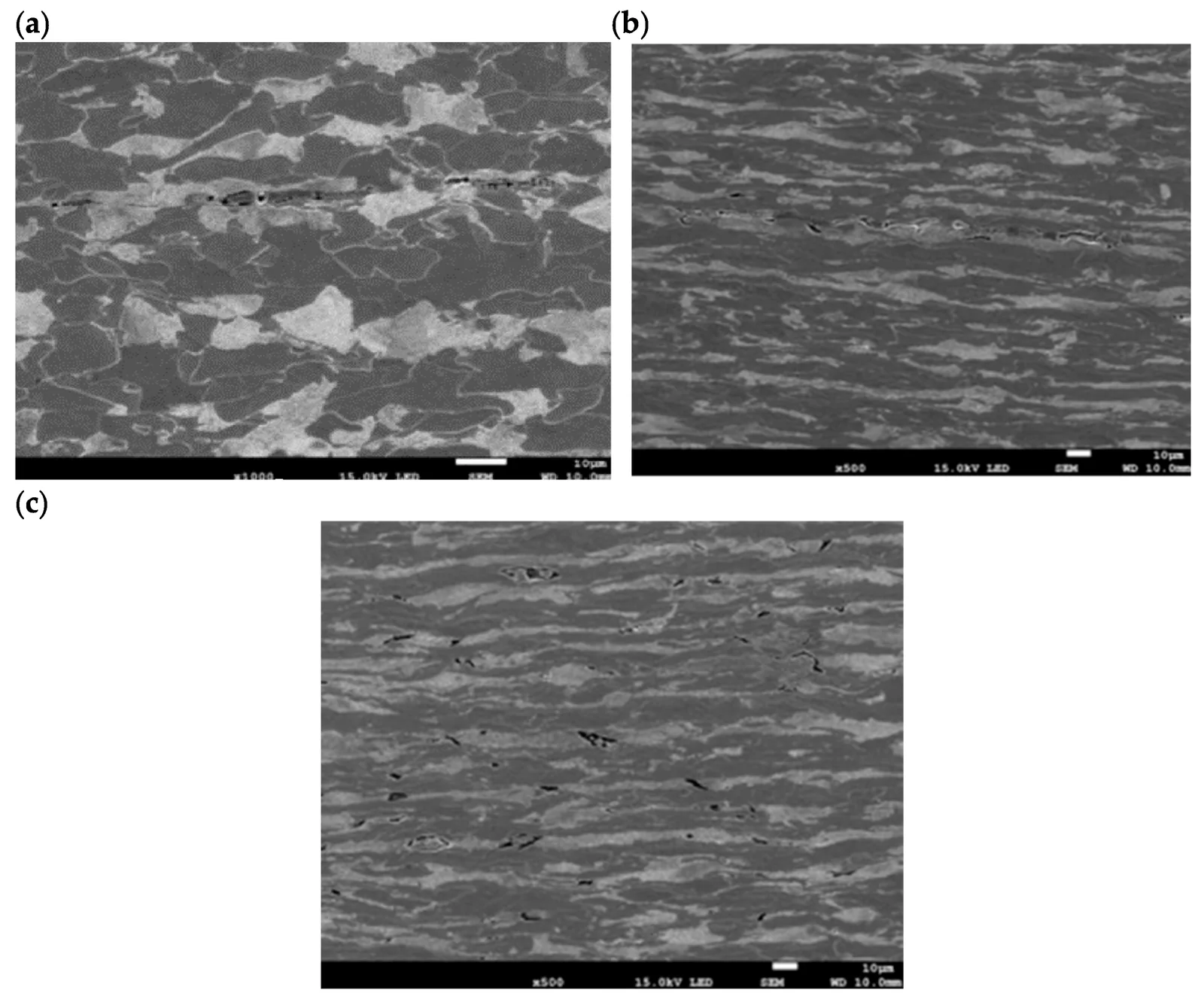
Microstructure of samples in the range of: (**a**) uniform deformation just before neck formation, (**b**) neck formation, (**c**) immediately before destruction.

**Figure 7:**
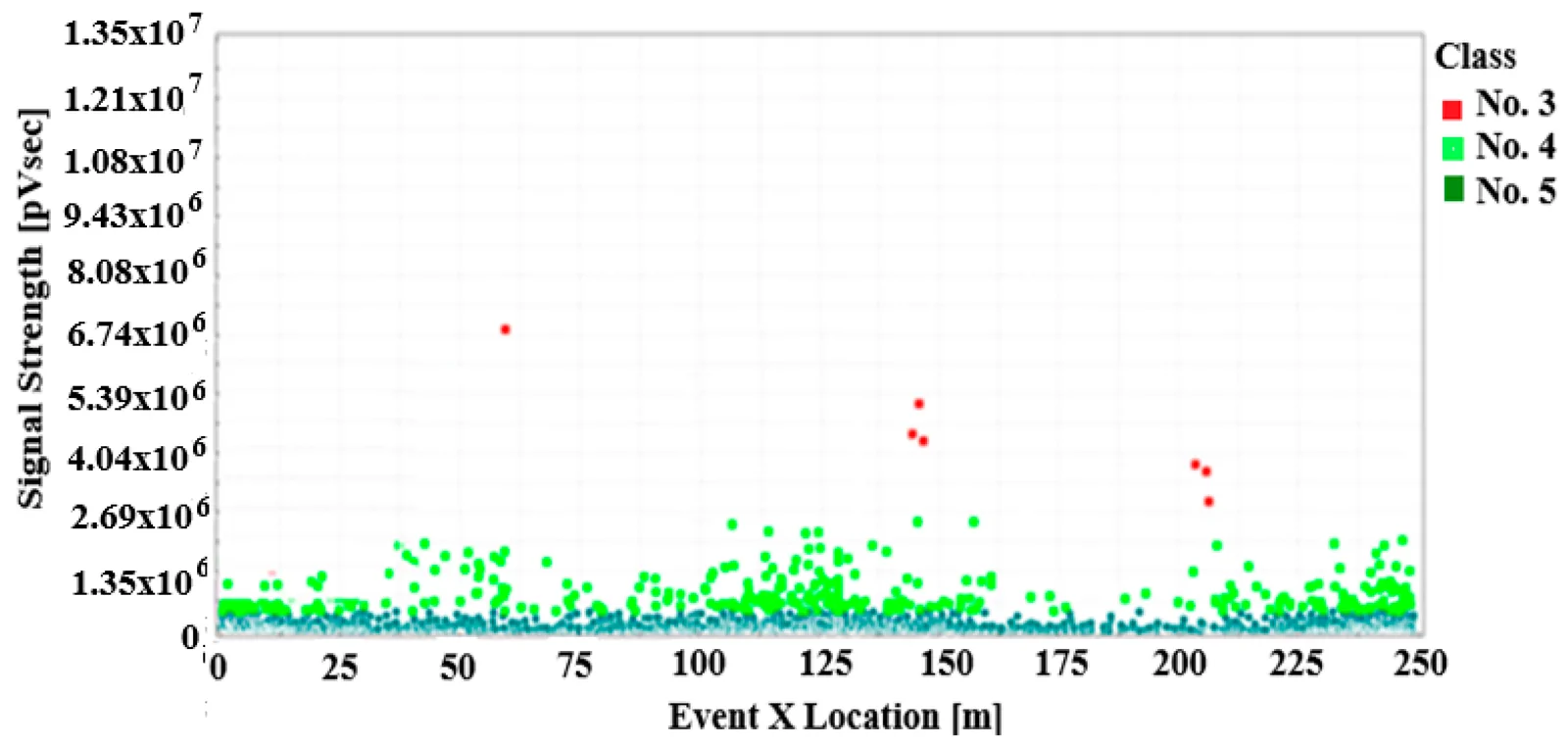
Spatial distribution of cumulative acoustic emission activity: Scatter plot demonstrating signal strength [pVsec] relative to linear source location [m] exported from the NOESIS 12.0 analytical programme.

**Figure 8:**
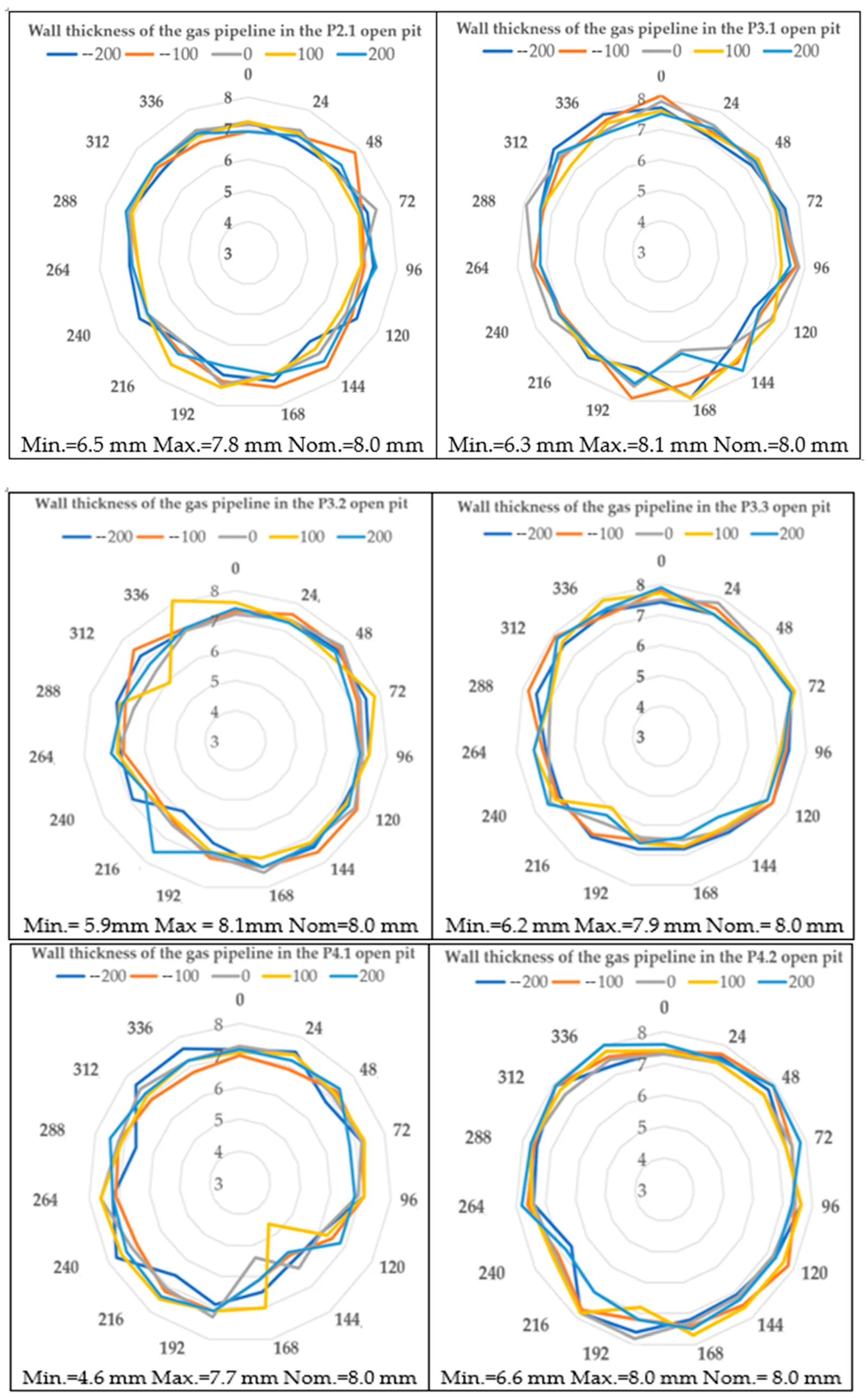
High-resolution pipeline wall thickness graphs with integrated spatial metrics obtained via 3D ultrasonic scanning at the designated control points and negative controls (All measurements explicitly detailed with millimeter [mm] units).

**Figure 9:**
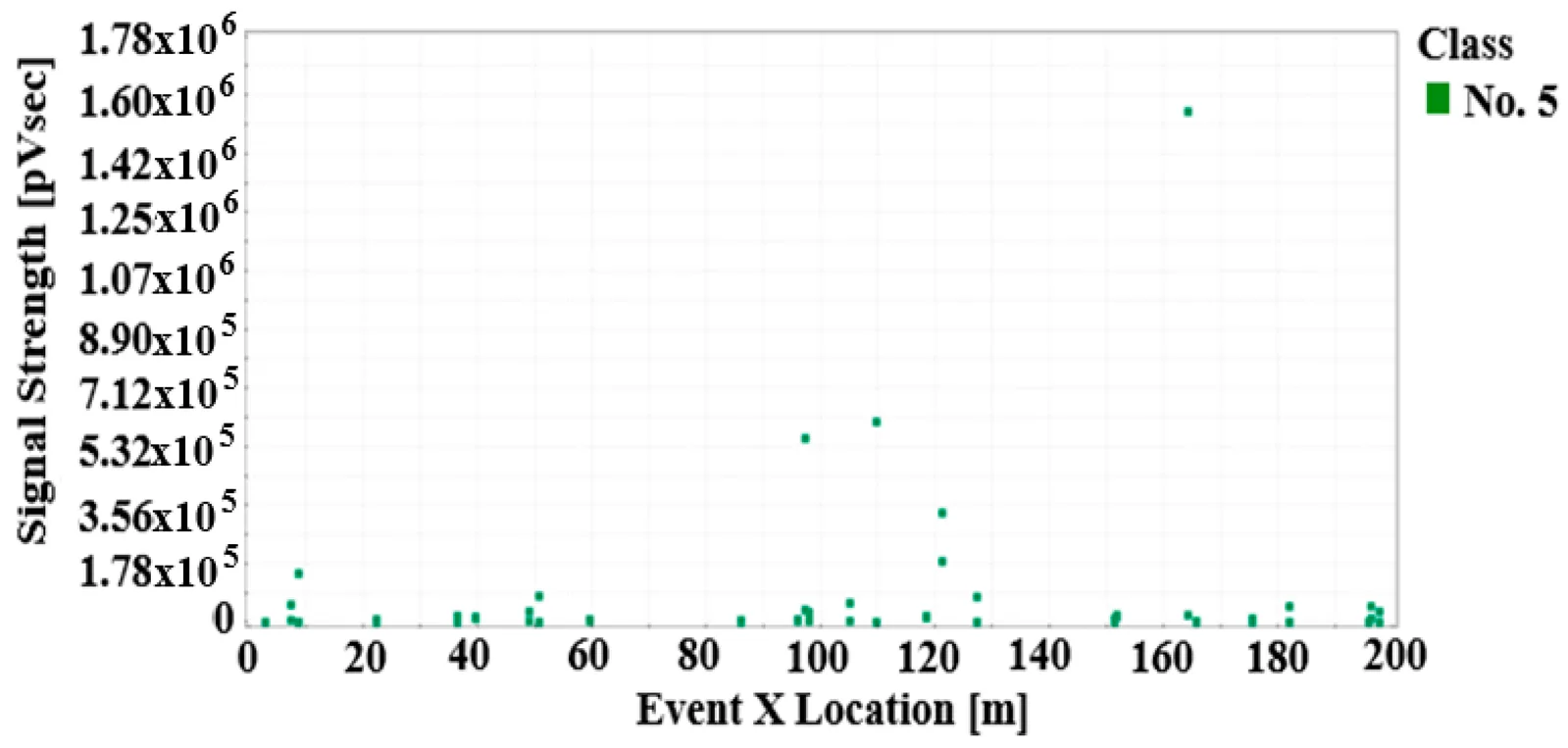
Spatial distribution of cumulative acoustic emission activity for the PE-KR-04 high-density polyethylene pipeline section: Scatter plot demonstrating absolute signal strength [dB] relative to linear source location [m].

**Table 1:** Anomaly classifications, graphical nomenclature, and structural hazard assessment levels.

Signal Class	Color Code	Unified Hazard Level	Interpretation of AE Signal Pattern and Associated Structural Condition
Class No 5	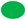	Low/Safe	AE signal pattern predominantly associated with background operational activity and low-level non-destructive structural or environmental sources.
Class No 4	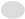	Moderate	AE signal pattern associated with lower-level anomalous activity; in selected validation cases, such patterns occurred in regions exhibiting limited wall-thickness reduction.
Class No 3	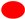	Moderate	AE signal pattern observed at locations where independent ultrasonic or metallographic examinations identified localized material loss or corrosion-related degradation.
Class No 2	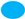	High/Critical	AE signal pattern associated in the investigated laboratory tests with advanced deformation and fracture-related activity.
Class No 1	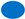	Catastrophic	AE signal pattern associated in the investigated experiments with severe structural degradation and, in selected validation cases, with high-amplitude signals observed during advanced fracture or leakage-related conditions.

**Table 2:** Geographical location, material characteristics, and revised technical designation of the monitored study sections.

No.	Designations	No.	Designations	No.	Designations	No.	Designations
1.	St-Knurów-DN200-1	6.	St-Tychy-DN300-1	11.	St-Rokitno-DN200-1	16.	PE-Zarki-DN160-1
2.	St-Lubania-DN300-1	7.	St-Siemian-DN300-1	12.	St-Rabka-DN150-1	17.	PE-Zarki-DN160-2
3.	St-Lubania-DN300-2	8.	St-Lipy-DN300-1	13.	St-Radk-DN200-1	18.	PE-Knurów-DN225-1
4.	St-Odrowąż-DN300	9.	St-Rabka-DN150-2	14.	St-Kędzierz-DN500-1	19.	PE-Zabrze-DN160-1
5.	St-Oświęcim-DN350	10.	St-Kędzierz-DN300-1	15.	St-Lub-DN225-1	20.	PE-Ruda Śl.-DN225-1

**Table 3:** Spatial localization coordinates, anomaly typologies, unified hazard levels, and control point designations.

No.	Control Point	Location	Type of Anomaly	Hazard Level	Anomaly Location [m]
1.	P2.1	GPS	Pitting	Moderate hazard	59.50 (9.50 from sensor 2)
2.	P3.1	GPS	Pitting	Moderate hazard	136.50 (38.50 from sensor 3)
3.	P3.2	GPS	Pitting	Moderate hazard	142.20 (44.20 from sensor 3)
4.	P3.3	GPS	Pitting	Moderate hazard	146.50 (48.50 from sensor 3)
5.	P4.1	GPS	Pitting	Moderate hazard	203.30 (3.30 from sensor 4)
6.	P4.2	GPS	Pitting	Moderate hazard	206.00 (6.00 from sensor 4)

**Table 4:** Classification Performance on the Evaluated AE Dataset.

Feature/Sensitivity	Class 1	Class 2	Class 3	Class 4	Class 5
Class 1 (Critical Failure)	185	10	5	0	0
Class 2 (Active Damage)	12	270	15	3	0
Class 3 (Pitting/Hazard)	2	18	315	15	0
Class 4 (Minor Anomaly)	0	4	22	1324	30
Class 5 (Safe Background)	0	0	10	574	7201

## Data Availability

The data presented in this study are available on request from the corresponding author.
